# RECRUITING SEXUAL AND GENDER MINORITY OLDER ADULTS WITH AND WITHOUT DEMENTIA IN LONG-TERM CARE: OVERCOMING BARRIERS

**DOI:** 10.1093/geroni/igae098.0799

**Published:** 2024-12-31

**Authors:** Morgan Wright, Tetyana Shippee, Tricia Skarphol, Jason Flatt, Andrew Alberth, Rajean Moone, B R Simon Rosser

**Affiliations:** University of Minnesota, Minneapolis, Minnesota, United States; University of Minnesota School Of Public Health, Minneapolis, Minnesota, United States; University of Minnesota, Minneapolis, Minnesota, United States; University of Nevada Las Vegas, Las Vegas, Nevada, United States; University of Massachusetts Boston, Boston, Massachusetts, United States; University of Minnesota, St. Paul, Minnesota, United States; University of Minnesota, Minneapolis, Minnesota, United States

## Abstract

Sexual and gender minority (SGM) older adults, including those using long-term care and living with cognitive impairment, are a growing population, but little is known about their unique experiences as they are often missing from research. This study investigates barriers and facilitators for recruitment of SGM older adults utilizing long term care (LTC) into research studies. As part of a larger study on SGM policies in LTC, we used purposive sampling to select twenty assisted living and nursing home directors for qualitative interviews. Participants were assisted living and nursing home administrators in Minnesota, identified as part of a larger survey on SGM policies in LTC. Qualitative coding and partially inductive/deductive thematic analysis resulted in the generation of three themes. The themes include the challenge of identifying SGM residents in long-term care, a need to be mindful of discrimination against SGM residents by other residents which can also hinder self-identification, and special considerations in working with SGM residents with AD/ADRD. Several strategies to overcome these barriers exist. The themes all contained information for future researchers that work with older SGM adults in long-term care facilities who may have AD/ADRD. Understanding barriers to identifying participants, addressing discrimination, and crafting appropriate consent processes for those with AD/ADRD are all important points for researchers.

